# Molecular characterisation of isogenic taxane resistant cell lines identify novel drivers of drug resistance

**DOI:** 10.1186/1471-2407-14-762

**Published:** 2014-10-14

**Authors:** Juliet Kenicer, Melanie Spears, Nicola Lyttle, Karen J Taylor, Linda Liao, Carrie A Cunningham, Maryou Lambros, Alan MacKay, Cindy Yao, Jorge Reis-Filho, John MS Bartlett

**Affiliations:** Biomarkers and Companion Diagnostics, Edinburgh Cancer Research Centre, Crewe Road South, Edinburgh, EH4 2XR UK; Transformative Pathology, Ontario Institute for Cancer Research, MaRS Centre, 661 University Ave, Suite 510, Toronto, Ontario M5G 0A3 Canada; Tumour Profiling Unit, Department of Molecular Pathology, Institute of Cancer Research, London, UK; Division of Cancer Therapeutics, Institute of Cancer Research, London, UK; Department of Pathology, Memorial Sloan-Kettering Cancer Center, 1275 York Avenue, New York, NY 10065 USA

**Keywords:** Breast cancer, Taxane, MDR, Cell cycle

## Abstract

**Background:**

Taxanes such as paclitaxel and docetaxel are used successfully to treat breast cancer, usually in combination with other agents. They interfere with microtubules causing cell cycle arrest; however, the mechanisms underlying the clinical effects of taxanes are yet to be fully elucidated.

**Methods:**

Isogenic paclitaxel resistant (PACR) MDA‒MB‒231, paclitaxel resistant ZR75‒1 and docetaxel resistant (DOCR) ZR75‒1 cell lines were generated by incrementally increasing taxane dose in native cell lines *in vitro*. We used aCGH analysis to identify mechanisms driving taxane resistance.

**Results:**

Taxane resistant cell lines exhibited an 18-170 fold increased resistance to taxanes, with the ZR75-1 resistant cell lines also demonstrating cross resistance to anthracyclines. Paclitaxel treatment of native cells resulted in a G2/M block and a decrease in the G1 phase of the cell cycle. However, in the resistant cell lines, minimal changes were present. Functional network analysis revealed that the mitotic prometaphase was lost in the resistant cell lines.

**Conclusion:**

This study established a model system for examining taxane resistance and demonstrated that both MDR and mitosis represent common mechanism of taxane resistance.

**Electronic supplementary material:**

The online version of this article (doi:10.1186/1471-2407-14-762) contains supplementary material, which is available to authorized users.

## Background

Breast tumours exhibit a wide degree of heterogeneity and diversity at both the cellular and molecular level. The taxanes, paclitaxel and docetaxel, are used successfully to treat breast cancer, alone or in combination with other agents [1]. Taxanes act by interfering with the spindle microtubule dynamics of the cell causing cell cycle arrest followed by cell death [2]. A significant proportion of patients progress despite treatment with taxane containing chemotherapy and there is a pressing need for both novel therapeutic options for patients failing taxane therapy and predictive biomarkers to select patients likely to benefit. Overexpression of P-glycoprotein (PgP/MDR1) is one of the most recognised mechanisms causing taxane resistance [3, 4]. However, several other candidate predictive biomarkers have been proposed in recent studies (AKT/HER2/TLE3) [5–7], but to date no robust, predictive diagnostic assay for taxane benefit or resistance has emerged. Whilst data suggests some patients are intrinsically resistant to taxanes and others acquire resistance to taxanes as treatment advances there is insufficient understanding of the clinical mechanisms underlying taxane resistance to develop either rational novel therapeutic or diagnostic approaches to target taxane based chemotherapy.

Progress in “targeting” conventional therapeutics such as anthracyclines and taxanes has been slow and has been hampered, in part, by a lack of focus and understanding of the key molecular events that lead to drug response or resistance in the clinical setting. Without significant progress in identifying the key molecular pathways driving drug resistance *in vivo*, we run the risk of continuing to seek to identify novel drugs and molecular diagnostics in a stochastic and largely unfocused manner.

Genome wide profiling of breast tumours is a powerful tool that can be used to correlate tumour characteristics to clinical outcome in patients. Many extensive studies have proposed novel and molecular subtypes of breast cancer which may have clinical relevance [8–12]. However few, if any, have proven effective as a basis for either targeting existing treatments or identifying novel therapeutic approaches in the context of drug resistance.

The overall aim of this study was to generate isogenic taxane-resistant breast cancer cell lines and elucidate the mechanisms that are driving resistance to taxanes in a pre-clinical model system. The studies summarised here characterise taxane resistant cell lines derived by the incremental increase of paclitaxel or docetaxel dose. The results presented demonstrate the ZR75-1 resistant cell line harbour cross-resistance to anthracyclines. An aCGH profile demonstrated a loss of mitotic pathways in the resistant cell lines indicating a potential theranostic pathway.

## Methods

### Cell culture and reagents

The breast cancer cell lines MDA-MB-231 and ZR75-1 (ATCC, Cedarlane Laboratories Ltd, Burlington, Canada) were cultured as monolayer in DMEM supplemented with 10% foetal calf serum, 10 mM glutamine and penicillin and streptomycin. Paclitaxel (Sigma, Oakville, Canada), docetaxel (Sigma, Oakville, Canada), epirubicin (Sigma, Oakville, Canada), doxorubicin (Sigma, Oakville, Canada) and carboplatin (Sigma, Oakville, Canada) were dissolved in dimethyl sulphoxide (DMSO) (Sigma, Oakville, Canada). Concentrated stock solutions were stored at -20°C. Drug resistant isogenic daughter cell lines were derived by incremental increases in drug concentrations over time until a stable taxane resistant phenotype was acquired. Cells were in each concentration of drug for two passages and until confluent, this ranged between 1-4weeks dependent on the dose. The following isogenic sub-lines were selected for further characterisation alongside each parent line: MDA-MB-231 25nM and 50nM paclitaxel resistant (MDA-MB-231 25PACR and MDA-MB-231 50PACR), ZR75-1 25nM and 50nM paclitaxel resistant (ZR75-1 25PACR, ZR75-1 50PACR) and 25nM and 50nM docetaxel resistant (ZR75-1 25DOCR, ZR75-1 50DOCR).

### IC50 and proliferation rates of parental and isogenic drug resistant lines

Dose response curves were set up by treating cells with increasing doses of the appropriate taxane: 0, 0.3, 1, 3, 10, 30, 100, 300, 1000 or 3000nM of either paclitaxel or docetaxel. Cross resistance to epirubicin, doxorubicin and carboplatin was assessed in a similar manner. Cell suspensions (100μl) were seeded in triplicate at a density of 30,000 cells/ml in 96 well plates and grown for 24 hours, washed and treated with drug for 72 hours. After 72 hours 100μl of growth media containing 10μl of CCK8 (Promega, Madison, USA) was added to each well for 3 hours at 37°C. The plates were then shaken for 10 minutes and optical density (OD) recorded at 450nm. IC50s were calculated using GraphPad Prism 5 (San Diego, USA). Stability of taxane resistance in MDA-MB-231 25PACR was assessed by maintaining the cells for 6 months with or without paclitaxel added to the growth medium. MDA-MB-231 parental cells were maintained without paclitaxel for an equivalent period for comparison.

### Flow cytometry

For cell cycle and DNA content analyses, native and resistant cells were plated in equal numbers into 6-well plates and synchronized by serum starvation overnight. Cells were then incubated with the appropriate concentration of taxane (25nM or 50nM of either docetaxel or paclitaxel), DMSO control or media alone control. The cells were collected after 24 and 48 hours, fixed with 80% ethanol and incubated with 2mg/ml RNase A (Sigma, Oakville, Canada) and 0.1mg/ml propidium iodide (Sigma, Oakville, Canada) for 30 minutes prior to analysis by flow cytometry. Data was collected by FACS Canto II and FACS Diva (both from BD Biosciences, Mississauga, Canada), and analyzed by FlowJo (Treesta, San Carlos, USA).

### DNA extraction and sample preparation for array Comparative Genomic Hybridisation

DNA was extracted from cells using the Qiagen Blood and Cell Culture Maxi kit (Qiagen, Toronto, Canada). DNA was stored in TE buffer pH 8.0 at 4°C.

### Microarray CGH

Cell line DNA was analysed on the Breakthrough Breast Cancer human CGH 4.6K 1.12 arrays as previously described [13]. Briefly, 1 μg of test and normal female genomic DNA, from pooled donor samples, was directly labelled with Cy3-dCTP or Cy5-dCTP (Amersham BioSciences, Amersham, UK) using a Bioprime labelling kit (Invitrogen, Paisley, UK) according to the manufacturer's protocol modified to incorporate 1.0 mM Cy dye, 0.6 mM dCTP, and 1.2 mM dATP, dGTP and dTTP. Unincorporated nucleotides were removed with MinElute purification columns (Qiagen, Crawley, UK). The labelled DNA was co-precipitated with 100 μg of Cot1 (Invitrogen, Paisley, UK), resuspended in hybridization buffer [50% formamide, 10% dextran sulphate, 2× SSC, 2% SDS, 2 mg of yeast tRNA (Invitrogen, Paisley, UK)], denatured at 75°C for 5 min, and pre-annealed for 30 min at 37°C. Slides were blocked in 10% BSA–50% formamide solution at 42°C for 45 min. The probe was subsequently applied to the slide and hybridized overnight at 42°C. Slides were washed in 2× SSC, 0.1% SDS for 15 min at 45°C; 2× SSC, 50% formamide for 15 min at 45°C; 2× SSC, 0.1% SDS for a subsequent 30 min at 45°C; and finally two 15-min washes of 0.2× SSC at room temperature. Slides were centrifuged at 1200 rpm for 2 min to dry. Each experiment was performed in duplicate as a dye swap to eliminate any labelling bias.

### Image acquisition and data analysis

Slides were scanned using an Axon 4000B scanner (Axon Instruments, Burlingame, CA, USA) and images were analysed using Genepix Pro 4.1 software (Axon Instruments). The median localized background slide signal for each clone was subtracted and each clone Cy5/Cy3 ratio subjected to print-tip loess normalization [14]. Dye swap experiments were collated, bacterial artificial chromosome (BAC) clone replicate spots averaged, and clones with poor reproducibility between replicates excluded (standard deviation >0.2).

### Network-based analysis

To examine whether genes showing common copy number gains or copy number losses across all three cell lines belong to a specific pathway, we conducted functional analysis of the common genes using Cytoscape Reactome Functional Interaction (FI) plugin in Cytoscape 3.0.2 (2013 FI network version). Genes were loaded using the gene set format with FI annotations and linker genes. Spectral clustering was performed to identify distinct network modules and subsequent pathway enrichment was calculated. Symbols were loaded as a gene set and interactions from the FI network 2012 version, including FI annotations and linker genes. Network modules were identified using spectral clustering and Pathway Enrichment computed for each module using the Reactome FI plugin functions. Reactome pathways exhibiting FDR values < 0.01 were considered enriched.

### MDR Resistance: RNAi Transfection of ZR75-1 resistant cells

A total of 2.6 × 10^5^ ZR75-1 25PACR cells were transfected with Lipofectamine RNAiMAX (Invitrogen, Paisley, UK) and siRNAs (each 30nM, Dharmacon, Waltman, USA) targeting MDR1, according to manufacturer’s instructions. As controls, transfection reagents without siRNAs were added (mock transfection) and cells were transfected with siRNA targeting GAPDH. After 48h cells were lysed for RNA analysis and 72h cells were lysed for protein analysis. The differences in IC_50_ were analysed and calculated as described above.

### Western blot analysis

Total protein lysates (20 μg) were separated by SDS-PAGE according to standard protocols [15] and immunoblotting was carried out using antibodies directed against PgP-specific MDR1 (G-1) (Santa Cruz Biotechnology, Santa Cruz, CA, USA) diluted 1:1000, GAPDH (14C10) (Cell Signalling, Whitby, Canada) diluted 1:5000 and β-actin (Calbiochem, La Jolla, USA) diluted 1:10000. Horseradish peroxidase–conjugated secondary antibodies were detected by ECL chemiluminescence (Amersham Biosciences, Plc.).

## Results

### Taxane resistant cell lines IC_50_s and cross resistance

The taxane resistant cell lines exhibited 18-170 fold increased resistance to taxanes, when IC_50_s were compared to those from parental cell lines, with cross resistance to both forms of taxane observed in all cell lines (Table 1). All ZR75-1 PACR and DOCR cell lines exhibited cross resistance to anthracyclines (epirubicin and doxorubicin); however, no cross-resistance was observed with carboplatin. MDA-MB-231 PACR cells were not cross-resistant to either anthracyclines or carboplatin (Table 1). 

**Figure 1:**
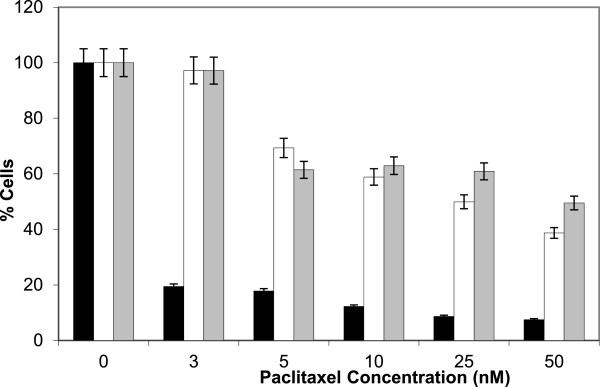
**MDA-MB-231 25PACR cells maintained resistance to paclitaxel after prolonged culture without exposure to taxane.** MDA-MB-231 25PACR cells were separated into two groups: one maintained and passaged, as normal in the presence of paclitaxel (white bar), the other was maintained and passaged in the absence of drug (grey bar)for a period of six months. The native cells are represented by the black bar. Cells were incubated with varying concentrations of paclitaxel and cell viability determined by CCK-8 assay. The X axis shows the increasing paclitaxel concentration measured in nM. The Y axis represents the percentage of cells with untreated cells being used as a baseline of 100%.

Following long term (6 months) culture of MDA-MB-231 25PACR cells in the absence of drug, cells were re-challenged with taxanes and the responses compared to parental and resistant cells cultured in the presence of taxanes (Figure 1). A two way Anova analysis of the proliferation data between the native and resistant cells with or without paclitaxel was performed in a pairwise fashion. When the two resistant cell lines were compared there was no significant difference between the two lines (p = 0.09728), indicating that they exhibited a very similar paclitaxel resistant phenotype.

**Table 1:** **IC**_**50**_ **values (nM) for paclitaxel, docetaxel, epirubicin, doxorubicin and carboplatin in isogenic MDA-MB-231 and ZR75-1 cell lines**

	Paclitaxel	Docetaxel	Epirubicin	Doxorubicin	Carboplatin
MDA-MB-231	1.6	0.8	35.15	35.64	208
MDA-MB-231 25PACR	29.61	6.4	34.25	61.38	212
MDA-MB-231 50PACR	89.98	10.16	30.39	30.12	266
ZR75-1	2.76	3.1	16.96	24.18	342.6
ZR75-1 25PACR	470.8	134.3	330.7	324.2	342.8
ZR75-1 50PACR	489.1	489.1	318.7	224.4	369.2
ZR75-1 25DOCR	41.24	42.13	3516	255.5	408.8
ZR75-1 50DOCR	310.1	47.24	506.9	676.7	386.9

### Cell cycle specific effects of taxanes

**Figure 2:**
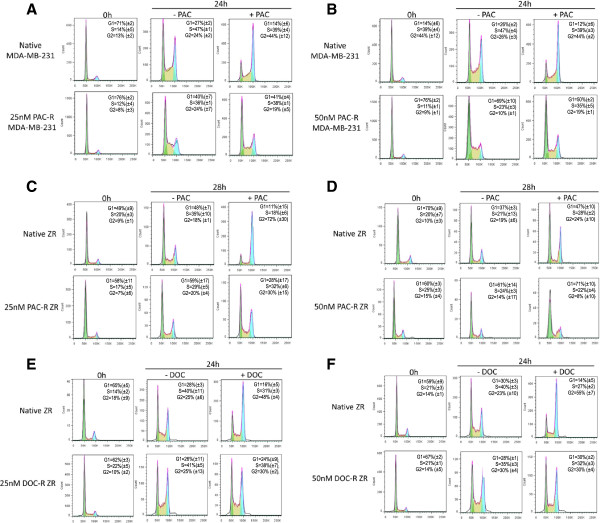
**Cell cycle analysis of native and resistant MDA-MB-231 and ZR75-1 by flow cytometry after synchronisation.** The native and respective resistant cell lines were treated with 25nM or 50nM of paclitaxel. The DNA content was measured by flow cytometry to determine the distribution of cell in each phase. The histograms demonstrate the cell cycle distribution within the cell population. **A** MDA-MB-231 native and MDA-MB231 25PACR cells with or without 25nM paclitaxel. **B** MDA-MB-231 native and MDA-MB231 25PACR cells with or without 50nM paclitaxel. **C** ZR75-1 native and ZR75-1 25PACR cells with or without 25nM paclitaxel. **D** ZR75-1 native and ZR75-1 25PACR cells with or without 50nM paclitaxel. **E** ZR75-1 native and ZR75-1 25DOCR cells with or without 25nM docetaxel. **F** ZR75-1 native and ZR75-1 50DOCR cells with or without 50nM docetaxel. Standard deviation of three experiments are shown in brackets.

Paclitaxel treatment of native MDA-MB-231 and ZR75-1 cells resulted in a G2/M block, and a failure to return to the G0/G1 phase (Figure 2). The G2/M population of the MDA-MB-231 native cells increased significantly from 24% to 44% upon paclitaxel exposure compared with a minimal change of 24% to 19% in the MDA-MB-231 25PACR cells. The increase of cell population at the G2/M phase was accompanied by a decrease of cell population in the G1 phase of the cell cycle for the native cells; however the resistance cell lines exhibited no change in the percentage of cells in the G1 phase. Paclitaxel treatment of native ZR75-1 cells resulted in a significant increase in the G2/M population from 18% to 72% and a decrease in the G1 population from 48% to 11%. While in the ZR75-1 25PACR cells there were minimal changes in the G2/M population from 20% to 30%, there was a decrease in the G1 population of cells from 59% to 28%. Treatment of the ZR75-1 50PACR cells with paclitaxel caused a slight decrease in the G2/M population of cells from 14% to 8% and a slight change in the G1 population of cells from 61% to 71% (Figure 2).

### Array comparative genomic Hybridisation

#### MDA-MB-231

**Figure 3:**
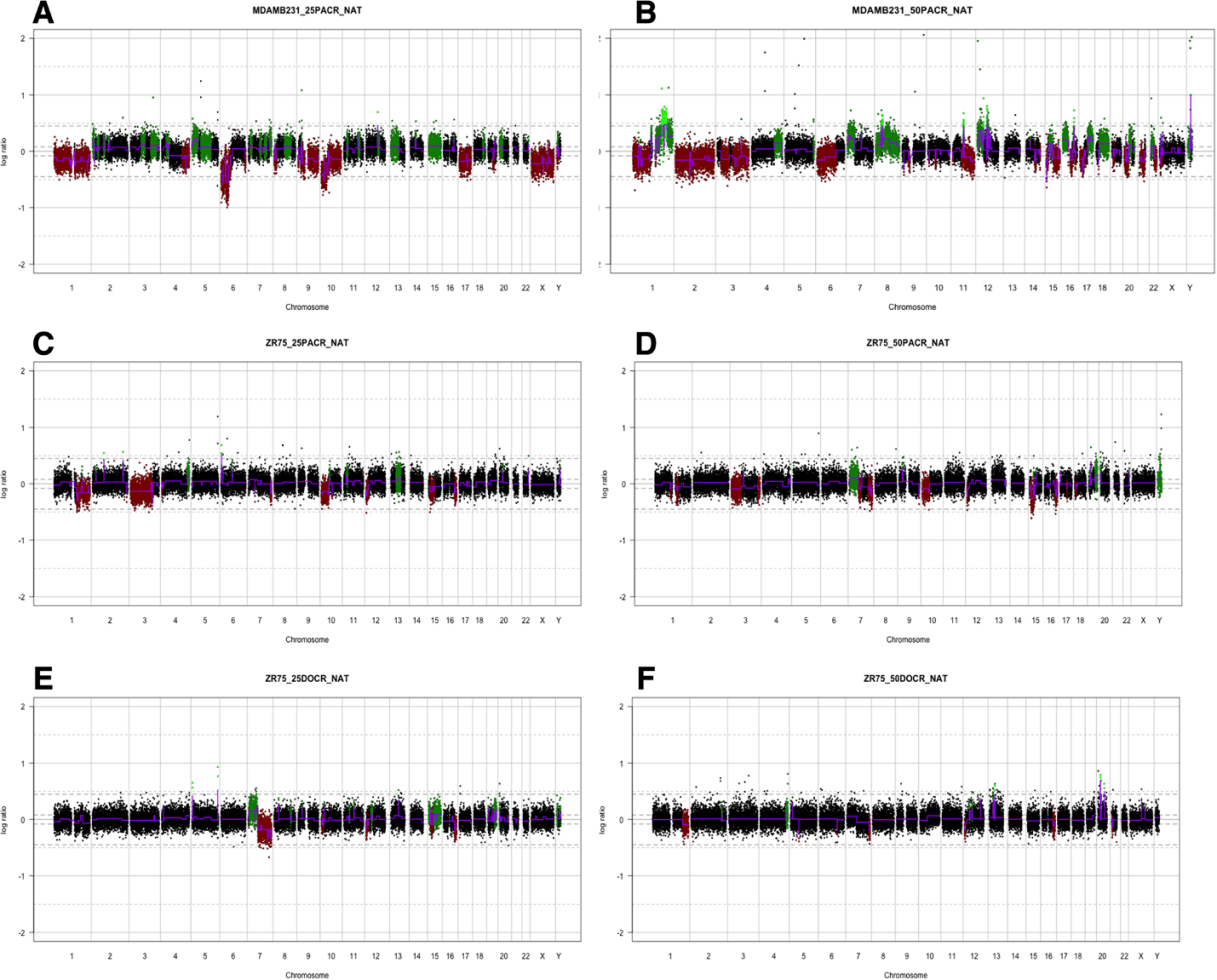
**aCGH of taxane resistant cell lines.** The plots show Log2Ratios of test to reference signal intensity from BAC clines in an aCGH experiment using DNA from native cells as a reference samples and DNA from resistant cells as a test sample. Navy dots represent BAC clones which remain unchanged, the green dots represent the BAC clones in which there is an area of gain on the genome, and the red dots represent the BAC clones in which there is an area of loss of the genome. The Log2ratio is measured on the Y axis and on the X axis the genome runs in chromosome order from 1 to the sex chromosomes. The p or short arm on each chromosome is followed by the q or long arm. The dotted lines represent the position of the centromere. The cbs algorithm recursively split chromosomes into segments based on the maximum t statistic estimated by each permutation (re Mathworks.com). **A.** MDA-MB-231 Natives vs MDA-MB-231 25PACR. **B.** MDA-MB-231 Natives vs MDA-MB-231 50PACR. **C.** ZR75-1 native cells vs ZR75-1 25PACR. **D.** ZR75-1 native cells vs ZR75-1 50PACR. **E.** ZR75-1 native cells vs ZR75-1 25DOCR. **F.** ZR75-1 native cells vs ZR75-1 50DOCR.

The PACR cell lines were analysed and compared to the parental controls (Figure 3A and B). Both the 25PACR and 50PACR cells demonstrated marked gains and losses (Table 2 and Figure 3A and B). There are three common areas of genomic loss in the MDA-MB-231 cell lines that extend with increasing paclitaxel resistance in chromosome 1p, 6p and 17p. Common areas of gain include 8q and 15p.

**Table 2:** **Common areas of loss, gain, deletion and amplification identified by aCGH in MDA-MB-231 PACR, ZR75-1 PACR and ZR75-1 DOCR at the two resistance levels 25nM and 50nM when compared to the native cell line**

Cell line	Extending loss	Extending gain	Deletion	Amplification
**231 PACR**	1p36.13-q44	2p25.3-23.3	6p21.1	6p21.1
	6p25.3-q12	3p24.3-q13.3	2q13	1q32.3
	8p	4p16.1-q12	15q11,2	4q21.21-21.22
	10p	5q14.3-q31.1	16 q11.2	8p12, 8p11.21
	19q	8q21.13-24.3		11q13.2
	X Chr.	11q15.1-q25		12q14.1
		centromeric 12		12q14.2
		centromeric 14		12q15
				15q11.2
				15q22.2-q22.3
**ZR75 PACR**	1q	None	None	None
	3p			
	7q			
	12p			
	15p			
	16q			
**ZR75 DOCR**	7q	None	None	None
	12p			
	16q			

#### ZR75-1

In the ZR75-1 cell lines there were fewer genomic changes that occurred once cell becomes resistant in contrast to the MDA-MB-231 (Table 2, Figure 3C-F). There were common areas of gains and losses in the 25PACR and 50PACR cells; losses were observed in 3p, 7q, 10p, 12p and 15p. Interestingly within region 7p22.3-q11.21 the gene ABCB5, a member of the p-glycoprotein family, is present and appears to be gained. There were no common areas of gain in the ZR75-1 PACR cell lines.

25DOCR and 50DOCR cells compared with native cells show area of loss in 7q, 12p and 16q again there were no common areas of gain with the DOCR cell lines.

When comparing the data obtained from the PACR and DOCR ZR75-1 cells the sole areas of common genomic alterations were losses at 7q and 12p.

#### Combined analysis

**Figure 4:**
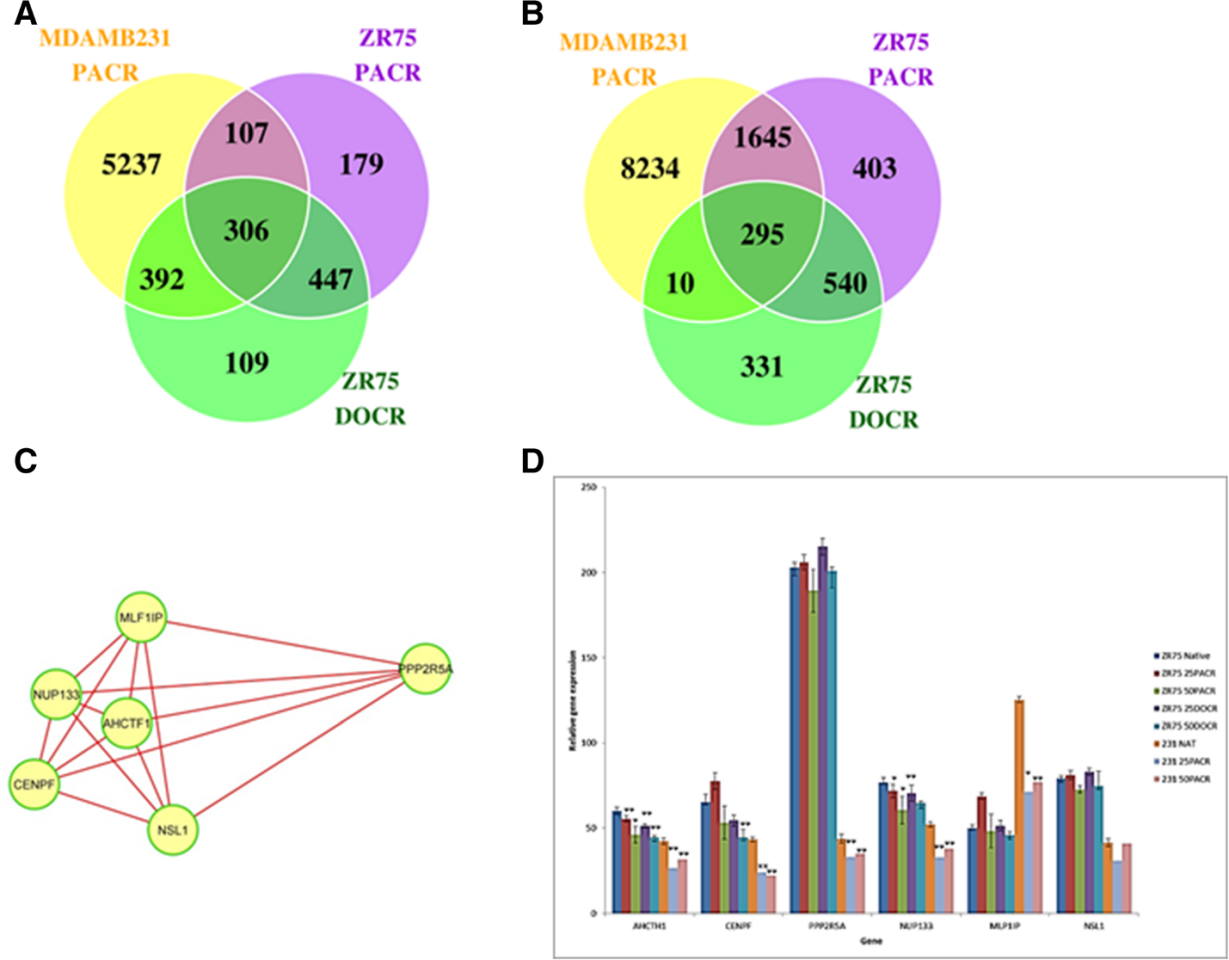
**Network-based analysis of MDA-MB-231 25PACR, ZR75-1 25PACR and ZR75-1 25DOCR taxane resistant cell lines. A.** Venn diagram of genes within significant areas of gain. **B.** Venn diagram of genes within significant areas of loss in 3 cell lines. **C.** Mitotic prometaphase module identified from functional interaction network analysis. **D.** Bar graph shows relative expression levels of AHCTH1, CENPF, PPP2R5A, NUP133, MLP1P and NSL1 in the MDA-MB-231 native, 25PACR, 50 PACR ZR75-1 25PACR, 50PACR and ZR75-1 25DOCR and 50DOCR taxane resistant cell lines. Error bars show standard deviation of three experiments. Asterisks indicate statistical difference * = p < 0.05, ** = p < 0.001.

When all areas of gain or loss across the 25nM resistant cell lines were combined, 295 known genes were identified as lost and 306 genes gained (Figure 4A and 4B). Following network analysis, eight modules were identified that contained significantly enriched pathways with a False Discovery Rate (FDR) <0.01. Each module contained clusters of connected genes. Module II contained 6 genes involved in the mitotic prometaphase. Interestingly, all six genes were deleted in taxane resistance cells and directly interconnected without linker genes (Figure 4C). These findings would suggest that loss of mitotic prometaphase regulatory genes is a common event associated with taxane resistance in breast cancer cells.

#### qRT-PCR validation of aCGH

qRT-PCR analysis was performed on the six deleted genes present in the resistant cell lines compared to parental controls. As shown in Figure 4C in the MDA-MB-231 resistant cell lines all six of the genes were downregulated compared to the parental control cells. Within the ZR75 cell lines downregulation of all resistant cell lines was demonstrated with AHCTH1 and NUP133. MLP1IP showed a decrease in expression in the both DOCR and 50PACR cells compared to the natives while the 25PACR cells showed an increase in expression.

### MDR1 is a driver of taxane resistance in ZR75-1 cells only

**Figure 5:**
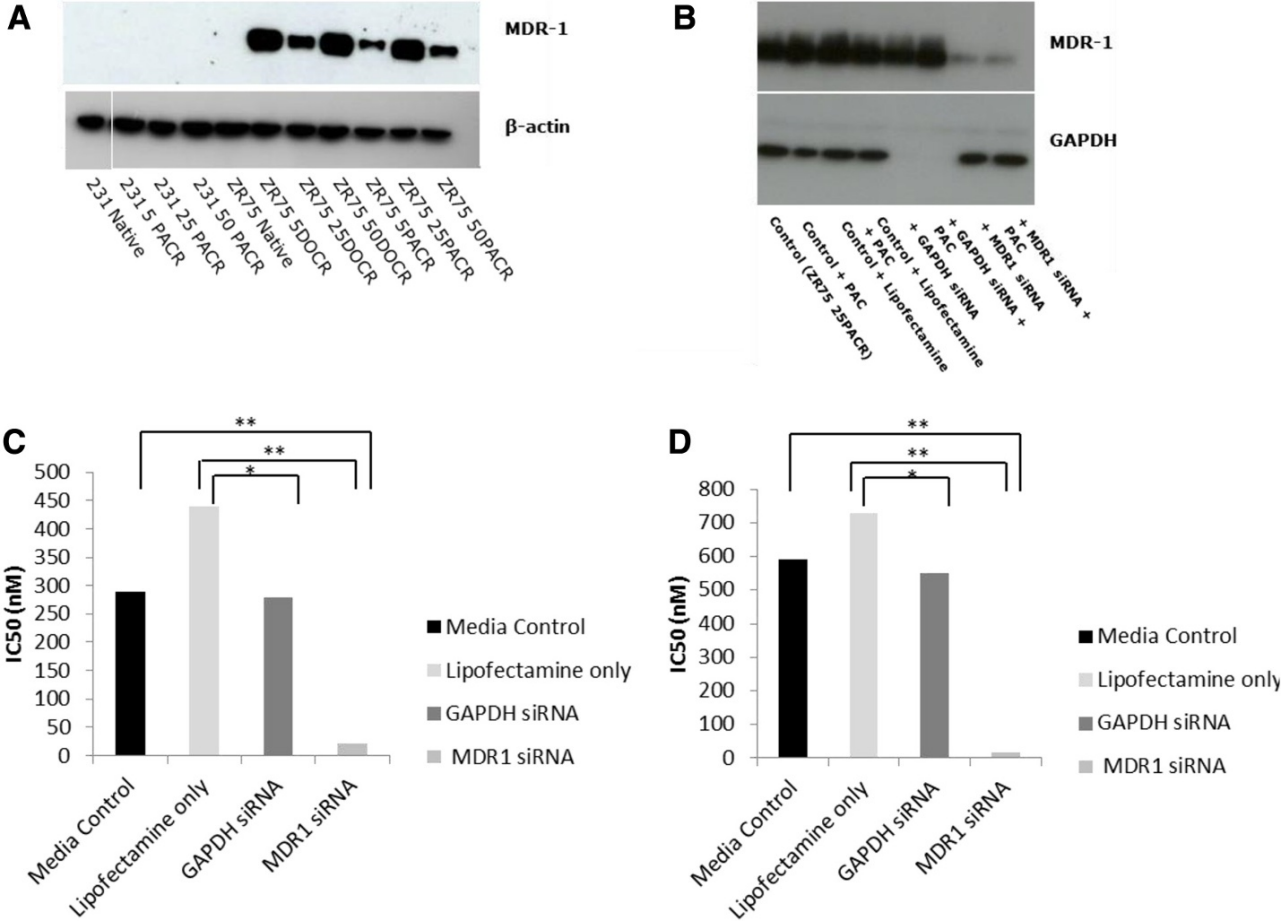
**Effects of MDR-1 knockdown in ZR75-1 paclitaxel resistant cells A Western blot analysis of proteins extracted from the cell lines and probed with MDR1. Actin was used as a loading control. B.** Western blot analysis of proteins extracted from ZR75-1 25PACR cells after transfections with MDR1 siRNA. Control cells were untreated, or with transfections reagents only or with siRNA targeting GAPDH; actin was used as a loading control. **C.** Graph shows the average IC_50_ of ZR75-1 25PACR cells after transfections with MDR1 siRNA. **D.** Graph shows the average IC_50_ of ZR75-1 50PACR cells after transfections with MDR1 siRNA. Asterisks indicate statistical difference * = p < 0.05, ** = p < 0.001.

No MDR‒1 protein expression was identified by western blotting in the MDA‒MB‒231 native, MDA-MB-231 25PACR or MDA-MB-231 50PACR cell lines (Figure 5A). There was a large increase in MDR1 protein expression in all four taxane resistant ZR75‒1 cell lines while no expression of the protein was observed in the ZR75‒1 native line. Western blot and cell proliferation assays were performed after down-regulation of MDR1 using siRNA. Western blot analysis demonstrated a reduction in MDR1 expression following transfection with siRNA (Figure 5B). In the proliferation assay MDR1 knock-down exhibited a 14- and 34-fold reduction in the IC_50_ concentration of paclitaxel in both the ZR75-1 25PACR and ZR75-1 50PACR cells respectively (Figure 5C and D). This corroborates previous western blot analysis suggesting MDR1 is the driver of taxane resistance in the ZR75-1 cell lines. There were no differences in α/β tubulin expression in either the ZR75 or MDA-MB-231 resistant cell lines compared to the native parental lines (Additional file 1: Figure S1). Taken together this would suggest taxane resistance in these cell lines is at least partially driven by MDR1 expression

## Discussion

The taxanes are a useful and effective group of chemotherapeutic agents that can be used as front line therapy to treat many types of cancer including breast, ovarian and prostate. Unfortunately, taxane resistance is a considerable clinical problem, and overcoming this is a key step to improving breast cancer patient survival. One way of combating this is to identify potential molecular drivers of taxane resistance so that they can be targeted with combination therapies to down-regulate the resistant phenotype.

In this study, a panel of isogenic paclitaxel resistant cell lines were generated by exposing parental cells to increasing concentrations of the appropriate taxane *in vitro*. We successfully generated daughter cell lines with markedly increased IC_50_s for taxanes; demonstrating clear resistance to these agents. Our cell cycle analysis demonstrated, in the native/parental cells, treatment with either docetaxel or paclitaxel resulted in a G2/M block. However, the drug resistant cell lines were able to overcome this G2/M block and progress through the cell cycle. Interestingly, we demonstrated that both the MDA‒MB‒231 and the ZR75‒1 native cell lines were more sensitive to docetaxel than paclitaxel which concurs with previous studies using other cell lines [16]. Other studies have suggested that docetaxel and paclitaxel affect different stages of the cell cycle with paclitaxel only targeting G2/M whilst docetaxel targets both S phase and G2/M [17]. In our hands treatment with both docetaxel and paclitaxel resulted in a G2/M block. Once reason that we may not see an S phase block is due to the concentration of drug that we were using. One study by Hernández‒Vargas used synchronized cells and then subjected them to low (2‒4nM) or high (100nM) concentrations of docetaxel. The low dose treatment caused a transient arrest and the high dose cause a prolonged arrest in mitosis. The short arrest leads to an aberrant mitosis and aneuploidy whereas the long arrest leads to mitotic slippage and tetraploidy [18]. A dual mechanism of cell cycle response has also been seen with paclitaxel treatment [19]. Low doses of paclitaxel have been shown to inhibit or retard the progression of mitosis and as a consequence alter microtubule dynamics rather than actually increasing polymer mass [20, 21]. At higher concentrations of paclitaxel cells become blocked in G2/M phase so that they cannot progress through mitosis.

Both ZR75-1 resistant cell lines showed cross-resistance to anthracyclines. This is consistent with a clinical trial of first-line treatment with anthracyclines followed by a crossover to taxanes which showed reduced response to taxanes [17], suggesting that anthracycline treatment may induce taxane cross-resistance. At a protein level both resistant ZR75-1 cell lines exhibited up-regulation of MDR1 suggesting that the resistance observed in these cells is mediated by the MDR family. The MDR family of p-glycoproteins are a common resistance mechanism observed in numerous *in vitro* studies. These proteins bind non-specifically to multiple chemotherapy drugs and actively export them across the cellular membrane [22, 23]. However, the clinical relevance of MDR genes remains to be elucidated. No cross-resistance was detected in the MDA-MB-231 PACR cells, consistent with previous studies performed in MCF-7 paclitaxel resistant cell lines [24] and suggests that a mechanism other than PgP glycoprotein is driving this resistance.

We performed a genomic analysis of our cell lines using aCGH. We compared each of the cell lines to DNA from pooled female blood and then compared each of the resistant lines with their respective native lines. Within the MDA-MB-231 cell, areas on chromosome 1p, 6p and 17p were lost and gains in chromosome 8q and 15p were observed. When comparing the native MDA-MB-231 cell line with the 50PACR cell lines, amplification at chromosome 1q was observed. Chromosome 1 aberrations are the most frequently described in a variety of cancers [25]. In breast cancer 1q gain is commonly observed across all subtypes, however the functional driver in this region has yet to be elucidated. There are many candidate genes; CENF, KIF14, DTL, NEK2, CKS1B, ASPM and EXO1 each of which are significantly associated with poor clinical outcome in breast cancer patients [26]. Interestingly, when functional network analysis was performed incorporating all three paclitaxel resistant cell lines, a signalling module which included genes controlling the mitotic prometaphase was identified. Five of the genes, PP2R5A, NUP133, AHCTF1, CENPF and NSL1, within this module are located on chromosome 1. Previously studies have demonstrated CENPF as both a prognostic and predictive gene in breast cancer [27]. One study showed CENPF to be associated with poor prognosis [28]. Paclitaxel enhances the stability of microtubules and mitosis is blocked at the metaphase-anaphase transition with prolonged blocking resulting in cell death. However, taxane resistant cells drug appear to have lost the ability to control this process. Drug resistant cells, when treated with taxane, progress through the cell cycle without arresting in G2/M suggesting they are bypassing a critical cell cycle checkpoint. Dysregulation of the mitotic metaphase check point is linked to chromosome instability (CIN). CIN cells become aneuploid and are associated with aggressive tumours and poor prognosis. CIN has been previously linked to taxane resistance in ovarian and colorectal cancer [29, 30]. Therefore, it would suggest that once these *in vitro* cell lines become taxane resistant they also become genomically unstable and therefore may be at greater risk of progression.

## Conclusions

In conclusion, our study has established a new model system to examine mechanisms of taxane resistance in breast cancer with genomic analysis showing a mitotic prometaphase as a predictor of resistance.

## Electronic supplementary material

Additional file 1: Figure S1: Western blot analysis of proteins extracted from the cell lines and probed with α/β Tubulin. GAPDH was used as a loading control. (DOCX 173 KB)
