# Supplementary figures and images for: Molecular characterisation of isogenic taxane resistant cell lines identify novel drivers of drug resistance
Source: BMC Cancer. 2014 Oct 14;14:762. doi: 10.1186/1471-2407-14-762 (PMC4203938; doi:10.1186/1471-2407-14-762)

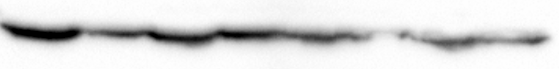

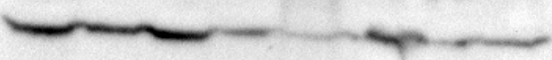


231 Native

231 25 PACR

231 50 PACR

ZR75 Native

ZR75 25DOCR

ZR75 50DOCR

ZR75 25PACR

ZR75 50PACR

α/β Tubulin

GAPDH

Supplement: Supplementary file 1 — Additional file 1: Figure S1: Western blot analysis of proteins extracted from the cell lines and probed with α/β Tubulin. GAPDH was used as a loading control. (DOCX 173 KB) [file 12885_2014_4941_MOESM1_ESM.docx]
